# Praktische Anwendung von Immunhistochemie in pankreatischen neuroendokrinen Neoplasien: Tipps und Pitfalls

**DOI:** 10.1007/s00292-023-01276-4

**Published:** 2024-01-04

**Authors:** Konstantin Bräutigam, Aziz Chouchane, Björn Konukiewitz, Aurel Perren

**Affiliations:** 1 Institut für Gewebemedizin und Pathologie, Universität Bern, Murtenstr. 31, 3008 Bern, Schweiz; 2 Institut für Pathologie, Universitätsklinikum Schleswig-Holstein, Campus Kiel, Christian-Albrechts-Universität zu Kiel, Kiel, Deutschland

**Keywords:** Neuroendokrin, Immunhistochemie, PanNET, Pankreas, Fallstricke, Neuroendocrine, Immunohistochemistry, PanNET, Pancreas, Pitfalls

## Abstract

Pankreatische neuroendokrine Neoplasien (PanNEN) sind eher selten. Die Morphologie hilft in der Zusammenschau mit der Immunhistochemie bei der Typisierung und weiteren Einteilung des jeweiligen Tumortyps. Je nach Tumorstadium und Differentialdiagnose variiert das diagnostische Panel. Die vorliegende Arbeit fasst die obligaten diagnostischen, prognostischen und prädiktiven Marker bei PanNEN zusammen.

Marker der Wahl zum Nachweis eines neuroendokrinen Phänotyps sind Synaptophysin, Chromogranin A sowie INSM1. Die Proliferationsfraktion Ki67 ist zur Graduierung unabdingbar, während p53 und Rb1 in der Abgrenzung zum neuroendokrinen Karzinom (NEC) helfen können. Transkriptionsfaktoren, wie beispielsweise CDX2, TTF‑1, Islet‑1 geben Hinweise auf die Lokalisation eines Primarius in der Cancer-of-unknown-primary(CUP)-Situation. Die DAXX/ATRX-Immunhistochemie hat vor allem prognostischen Wert. Molekularpathologische Untersuchungen haben bisher einen geringen Stellenwert in der Diagnostik der PanNEN.

Wichtiger Fallstrick in der Routinediagnostik ist das breite Spektrum an Differentialdiagnosen, welche neuroendokrine Neoplasien imitieren. Ein erweitertes immunhistochemisches Panel ist im Zweifelsfall empfohlen.

## Abstract (en)

Pancreatic neuroendocrine neoplasms (PanNEN) are rather rare entities. Morphology, combined with immunohistochemistry, allows typing and grading, thereby leading therapeutic decisions. Depending on tumor stage and differential diagnosis, a broad diagnostic panel may be required. The present work summarizes the minimal diagnostic, prognostic, and predictive markers in PanNEN.

Markers of choice for defining a neuroendocrine phenotype are synaptophysin, chromogranin A, and INSM1. The proliferation fraction Ki67 is indispensable for grading, while p53 and Rb1 can help in the differentiation from neuroendocrine carcinoma (NEC). Transcription factors, such as cdx2, TTF‑1, and Islet‑1, can indicate the site of a primary tumor in the setting of a cancer of unknown primary (CUP). DAXX/ATRX immunohistochemistry has mainly prognostic value. Molecular pathology studies currently have little practical value in the diagnosis of PanNEN.

An important pitfall in routine diagnostics is the wide spectrum of differential diagnoses mimicking neuroendocrine neoplasms. An expanded immunohistochemical panel is strongly recommended in case of doubt.

## Immunhistochemische Marker neuroendokriner Differenzierung

**Figure 1:**
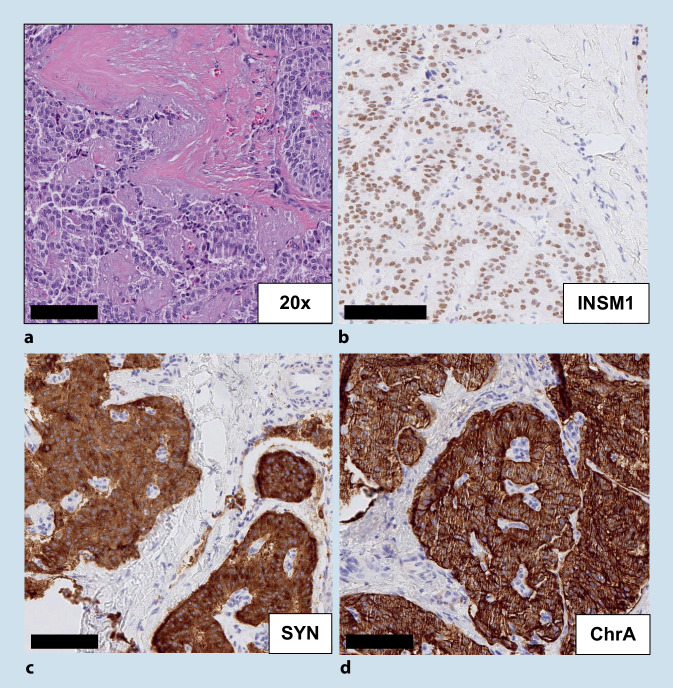

Gut und schlecht differenzierte pankreatische neuroendokrine Neoplasien (PanNEN) zeichnen sich durch die Expression der neuroendokrinen Marker Synaptophysin, Chromogranin A und INSM1 aus. Gut differenzierte PanNEN sind meist stark und durchgehend positiv für Synaptophysin, Chromogranin A und INSM1 (Abb. 1). Die Expression der neuroendokrinen Marker nimmt in schlecht differenzierten PanNEN ab. Chromogranin A ist häufig negativ, INSM1 und Synaptophysin können nur sehr fokal exprimiert sein [1]. Es gibt noch weitere Marker, die den neuroendokrinen Phänotyp erkennen, aber nicht spezifisch (CD56) oder schwierig einzustellen sind (NSE). Daher sollten sie nicht für die Diagnose neuroendokriner Neoplasien verwendet werden. Zudem können neuroendokrine Marker in konventionellen Adenokarzinomen exprimiert sein. Gemischte neuroendokrine-nichtneuroendokrine Neoplasien können erst dann diagnostiziert werden, wenn auch eine typische, morphologisch distinkte Komponente konventionell histologisch erkennbar ist [2–4].

## Proliferationsindex „Ki67“

Die Proliferationsfraktion Kiel 67 (Ki67) ist ein seit Langem etablierter Biomarker zur Gradierung pankreatischer neuroendokriner Neoplasien [5]. Wichtige Cut-offs sind 3 % und 20 %. Die Quantifizierung mittels Auszählung auf einem Ausdruck ist verlässlich und kostengünstig [6].

**Figure 2:**
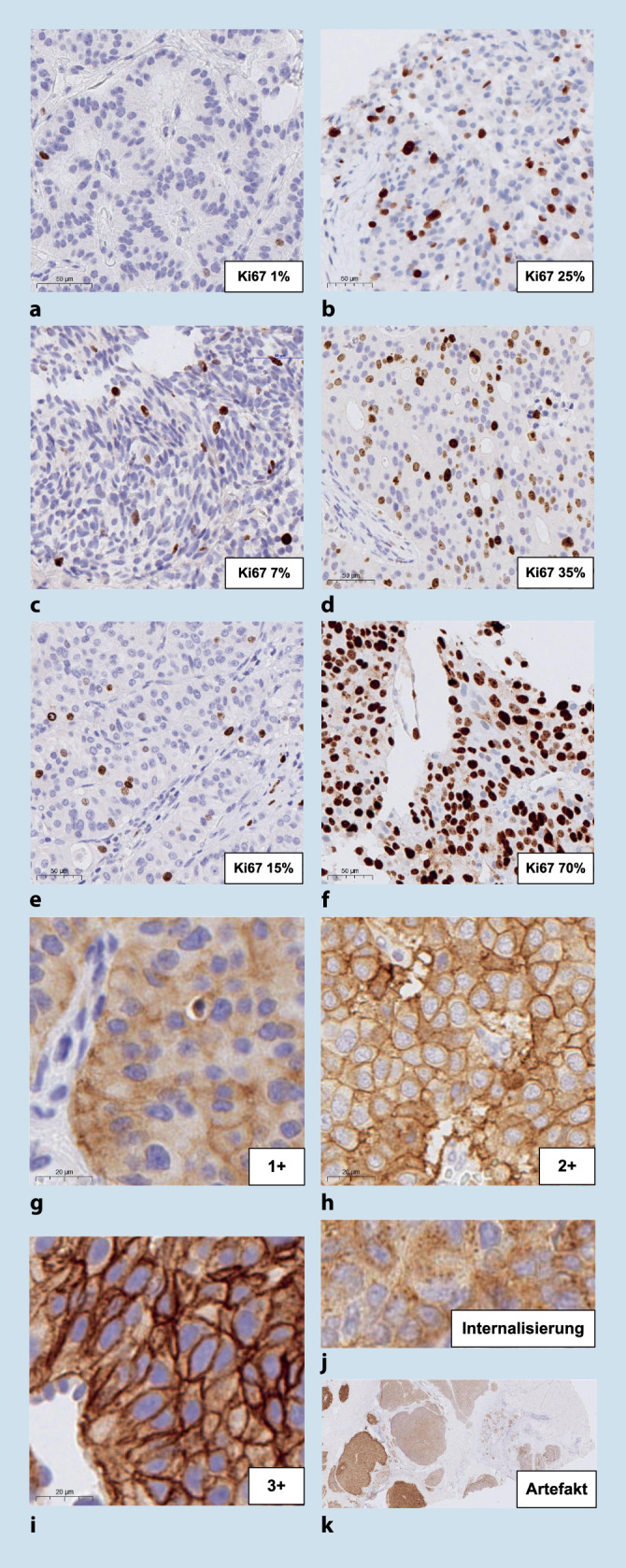

Ki67 wird im „Hotspot“ (mind. 500 Zellen) gezählt (Abb. 2) und kann auf Schnittpräparaten heterogen sein [7]. Eine PHH3-Immunhistochemie detektiert Mitosefiguren und ist weniger gut untersucht als Mitosefiguren in der HE-Färbung oder Ki67. Gastroenteropankreatische neuroendokrine Karzinome (NEC), G3, zeigen bei einem Ki67 < 55 % ein reduziertes Ansprechen auf platinbasierte Therapie [8].

## Somatostatinrezeptor 2A (SSTR2A)

Neuroendokrine Tumoren können Somatostatinrezeptoren (SSTR) exprimieren, die diagnostisch, prognostisch [9] und therapeutisch als Zielmoleküle relevant sind [10]. Somatostatinanaloga werden an radioaktive Isotope gekoppelt und binden so an SSTR (interne Peptidradiorezeptortherapie).

Der Nachweis von SSTR2A erfolgt immunhistochemisch. Die Expression ist membranär, allerdings heterogen und teils deutlich fixierungsabhängig (Cave: Fixierungsgradient als Artefakt, Abb. 2; [11]). Endothel kann als interne Positivkontrolle genutzt werden. Nichtfunktionelle pankreatische neuroendokrine Tumoren (PanNET) zeigen häufig eine starke Expression, während Insulinome häufig negativ sind. Eine Intensitätseinteilung in 3 Stufen hat sich bewährt (Abb. 2). Die Intensität und der prozentuale Anteil positiver Zellen (> 10 % membranäre Positivität) korrelieren mit der realen Rezeptordichte [12]. Zu beachten ist eine dosisabhängige SSTR2-Internalisierung bei Pankreasoperationen unter Einsatz von Somatostatinanaloga, bspw. Octreotid [13]. Dies ist nicht zu verwechseln mit einer schwachen Expression von SSTR2A.

## p53 und Rb1

**Figure 3:**
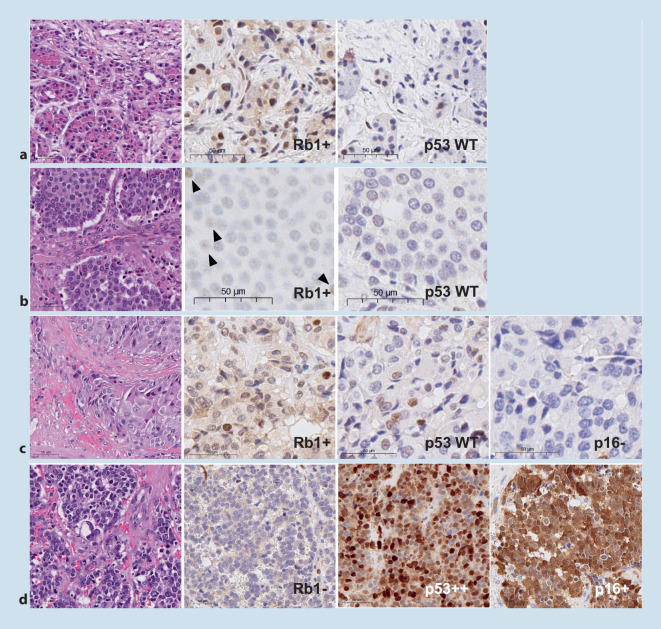

In der differentialdiagnostischen Abgrenzung von NET G3 zu NEC haben sich die beiden Marker p53 und Rb1 als hilfreich erwiesen. *RB1 *und *TP53* sind Tumorsuppressorgene, die in PanNET ganz überwiegend einen Wildtypstatus aufweisen und in bis zu 70 % der NEC alteriert sein können (Abb. 3). Das Expressionsmuster von Rb1 gilt im Falle eines kompletten nukleären Verlustes als pathologisch, die Endothelien der intratumoralen Blutgefäße dienen als interne Positivkontrolle. Dieser Rb1-Verlust ist häufig epigenetisch induziert. Ein Rb1-Verlust kann einen prädiktiven Wert für eine platinbasierte Therapie haben [14].

Die Interpretation des Expressionsmusters von p53 ist komplexer. Hier sind sowohl der komplette nukleäre Verlust als auch die nukleäre Überexpression pathologisch und mit inaktivierender Mutation vergesellschaftet. Allerdings muss man in einigen Fällen zum einen zur Abgrenzung eines kompletten nukleären Verlustes positive Tumorzellkerne regelrecht suchen, zum anderen existiert kein verlässlicher *Cut-off* zur Definition einer nukleären Überexpression, was in einigen Fällen zu diagnostischer Unsicherheit führen kann. Eine starke nukleäre Expression von p53 in mehr als 20 % der Tumorzellen korreliert gut mit einer *TP53-*Mutation. Die pathologische Expression von Rb1 und p53 ist ein indirekter Hinweis auf eine Genalteration, der Mutationsstatus ist allerdings nicht vorhersagbar, da auch Veränderungen der Genkopienzahl und epigenetische Regulationen eine entscheidende Rolle spielen. Die definitive Einordnung von NET G3 und NEC muss allerdings in erster Linie aufgrund histologischer Wachstumsmuster erfolgen, da auch wenige NET G3 eine pathologische Expression von p53 (10 %) und Rb1 (selten) zeigen können [1, 3, 15, 16].

## *DAXX/ATRX* und Menin

*DAXX* und *ATRX* codieren für Proteine, die die genomische Stabilität unterstützen, insbesondere in den Telomerregionen des Genoms. Somatische Mutationen in diesen Genen schließen sich gegenseitig aus und führen zu alternativer Telomerverlängerung (ALT). *DAXX*- und *ATRX*-Mutationen treten meist erst in größeren (> 2 cm) PanNET auf und sind ein Progressionsmechanismus.

Immunhistochemisch ist der nukleäre Expressionsverlust eines dieser Proteine mit einer schlechteren Prognose und einer höheren Rezidivrate bei PanNET assoziiert [15]. Endothel, Stroma oder Immunzellen können als interne Kontrollen verwendet werden, um falsch negative Befunde zu vermeiden.

*MEN1*-Mutationen sind die häufigsten Mutationen bei PanNET, nicht nur im Rahmen genetischer Syndrome, sondern auch bei sporadischen Tumoren. Der immunhistochemische Verlust der Menin-Expression, der auch im Zellkern zu beobachten ist, kann die Diagnose unterstützen. *MEN1*-Mutationen sind nicht mit schlechterer Prognose assoziiert.

## Bedeutung von Transkriptionsfaktoren

Transkriptionsfaktoren helfen bei der Bestimmung des Ursprungs einer neuroendokrinen Neoplasie. Islet‑1 ist spezifisch für einen pankreatischen Primarius (kann im Duodenum oder Rektum ebenfalls positiv sein) [17, 18]. PDX1 und ARX als Transkriptionsfaktoren für β‑ und α‑Zellen können im Zweifel einen pankreatischen Ursprung weiter erhärten (wie auch ein Expressionsverlust von DAXX/ATRX). Während „thyroid transcription factor 1“ (TTF-1) in erster Linie einen pulmonalen oder thyreoidalen (medulläres Schilddrüsenkarzinom) Ursprung anzeigt, deutet CDX2 auf einen gastroenteropankreatischen Primarius. PITX2 wurde kürzlich als nützlicher Adjunkt für neuroendokrine Primarien des Mitteldarms beschrieben [19]. Falls es sich bei dem Primarius um ein neuroendokrines Karzinom handelt, sind diese Transkriptionsfaktoren nicht aussagekräftig.

Eine Erweiterung der diagnostischen Immunhistochemie ist insbesondere bei zweifelhafter Morphologie und klinisch-bildgeberischer Korrelation geboten. Dies gilt speziell für Differentialdiagnosen, welche eine neuroendokrine Morphologie imitieren (siehe Tab. 1 sowie Beitrag im vorliegenden Themenheft).

**Table 1:** 

Fragestellung	Immunhistochemische Marker
Neuroendokrine Neoplasie?	Chromogranin A, CK, INSM1, Synaptophysin
DD Neuroendokrines Karzinom	DAXX/ATRX/MEN1, p16, p53, Rb1
Ursprung der neuroendokrinen Neoplasie	CDX2, DAXX/ATRX, Islet‑1, TTF‑1, PITX2, (ARX, PDX1)
MiNEN	BCL10, CA19.9, CEA, EMA, MUC1, Trypsin, ggf. weitere
Solid-pseudopapilläre Neoplasie (SPN)	β‑Catenin, Chromogranin A, CK
Metastase klarzelliges Nierenzellkarzinom	Chromogranin A, RCC (Islet-1/PAX8 können positiv sein)
Paragangliom	Zytokeratin negativ, GATA3
Azinuszellkarzinom	BCL10, Trypsin
Adrenokortikales Karzinom	SF1

*CK* Zytokeratin, *DD* Differentialdiagnose, *MiNEN* gemischt neuroendokrin-nichtneuroendokrine Neoplasien, *PAX8* „paired box gene 8“, *RCC* „renal cell carcinoma“, *SF1* „steroidogenic factor 1“

## Relevanz der Hormone

PanNETs können eine Vielzahl unterschiedlicher Peptidhormone produzieren, die neben den Hormonen der Langerhans-Inseln (Insulin, Glukagon, Somatostatin, pankreatisches Polypeptid) auch ektope Hormone einschließen (z. B. Serotonin, Gastrin, VIP, ACTH). In einigen Fällen ist die Hormonproduktion mit einem klinischen Syndrom assoziiert. Dann werden die Tumoren nach Ihrem produzierten Hormon benannt (u. a. Insulinom, Glukagonom, Gastrinom). Für die Routinediagnostik primärer PanNET ist der immunhistochemische Hormonnachweis nur in Ausnahmefällen relevant, da hormonaktive PanNET meist eine charakteristische hormonspezifische Klinik (Flush, Diarrhö, u. a.) zeigen, die den klinischen Kolleg*innen in Kombination mit laborchemischen Hinweisen in der Regel genügen. In der metastatischen Situation kann der immunhistochemische Nachweis von Peptidhormonen bei unbekanntem Primarius differentialdiagnostisch allerdings in vielen Fällen hilfreich sein [3, 20].

## Immunhistochemische Tricks bei Pitfalls

### NET G3 versus NEC

**Figure 4:**
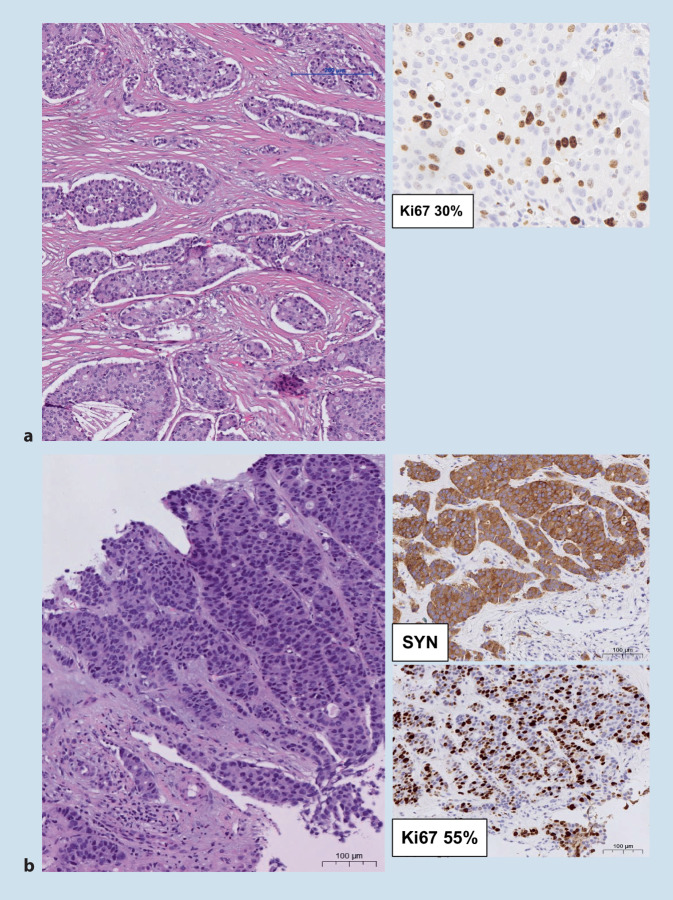

Die Unterscheidung zwischen NET G3 und NEC ist für die Wahl der Systemtherapie und die Prognose entscheidend. NET G3 entstehen im Pankreas häufiger als in anderen Organen des Gastrointestinaltrakts. Sowohl Histomorphologie, immunhistochemische und molekulare Untersuchungen als auch klinische Geschichte helfen bei der Unterscheidung ([14, 21]; Abb. 4, Tab. 2). Bei einem NET in der Vorgeschichte ist Vorsicht bei der Diagnosestellung eines NEC angebracht. In der seltenen Situation einer Transformation schlagen wir eine Bezeichnung als NET G3 mit „NEC-artigen Eigenschaften“, die klinische Bedeutung ist noch nicht geklärt, vor.

**Table 2:** 

	NET G3	NEC
*Wachstumsmuster*	Organoid	Expansive solide/kribriforme Areale
*Stroma*	Hyalinisiert	Desmoplastisch
*Kapilläre Gefäße*	Eng mit Nestern assoziiert	Entfernt von Gefäßen
*Nekrose*	Fehlend oder punktförmig	Geografisch
*Synaptophysin/Chromogranin A*	Homogen stark	Fleckförmig
*p53*	Pathologisch in 10 %	Pathologisch in 50 %
*Rb1-Verlust*	< 5 %	70 %
*p16-Überexpression*	Nicht vorhanden	70 % stark positiv (Achtung Zervix bei HPV)
*Verlust DAXX/ATRX/MEN1*	70 % von PanNET, in anderen NET weniger relevant	< 10 %
*Ki-67*	Häufig < 55 %	Häufig > 55 %

*PanNET* pankreatische neuroendokrine Tumoren

### NEC vs. MiNEN vs. Adenokarzinom mit neuroendokriner Differenzierung

Die Histomorphologie ist entscheidend für die Unterscheidung zwischen NEC, gemischten neuroendokrinen Neoplasien (MiNEN) und Adenokarzinomen mit neuroendokriner Differenzierung (ADNE).

Morphologische Adenokarzinome, die diffus Synaptophysin exprimieren, sind im Kolon gut untersucht und als ADNE beschrieben. Sie kommen selten auch im Pankreas vor. Neben der morphologisch erkennbaren drüsigen Komponente kann der fehlende Nachweis einer relevanten Expression von Chromogranin A und INSM1 helfen. Da zur Diagnose eines MiNEN mindestens 30 % beider Komponenten vorliegen müssen, empfehlen wir bei Biopsaten eine beschreibende Diagnose einer gemischten Neoplasie (Adenokarzinom und großzelliges NEC) mit Kommentierung, dass formell 30 % notwendig sind. Das gemischte azinär-neuroendokrine Karzinom als Subtyp einer MiNEN wird insbesondere als metastatische Läsion nicht selten als NET G3 verkannt.

### Anwendung von Molekularpathologie in pankreatischen neuroendokrinen Neoplasien

Molekulare Schlüsselalterationen in pankreatischen neuroendokrinen Neoplasien sind *MEN1*-Mutationen als initiierende Veränderung sowie *DAXX*- oder *ATRX*-Mutationen im Rahmen der Progression [22]. Eine Anwendung von DAXX/ATRX-Immunhistochemie hat sich als prognostischer Marker zusätzlich zu Ki67 bewährt. Die immunhistochemische Untersuchung von Menin sowie die Anwendung der Fluoreszenz-in-situ-Hybridisierung zur Detektion alternativer Telomerverlängerung (ALT) haben bislang keinen Eingang in die Routine gefunden. Next Generation Sequencing (NGS) kann in Einzelfällen zur Unterscheidung von NET G3 versus NEC bei inkonklusiver Morphologie und Immunhistochemie hilfreich sein, da NEC genetische Alterationen aufweisen können, die dem pankreatischen duktalen Adenokarzinom näherstehen.

In kolorektalen neuroendokrinen Neoplasien wurden Mikrosatelliteninstabilität sowie *BRAF*-Mutationen [23, 24] als therapeutisch relevant beschrieben. In PanNEN dagegen gibt es diesbezüglich keine klare Datenlage. Mausmodelle zeigen bei pankreatischen neuroendokrinen Tumoren verschiedene molekularpathologische Subtypen mit heterogenem metabolischen Profil und unterschiedlicher biologischer Aggression [25]. Reproduzierbare Indikatormoleküle für diese Subtypen sind bisher nicht publiziert.

## Fazit für die Praxis

- Synaptophysin, Chromogranin A und Insulinoma-assoziiertes Protein 1 (INSM1) sind etablierte Marker zur Determinierung einer neuroendokrinen Neoplasie.
- Die Proliferationsfraktion Ki67 ist unerlässlich zur WHO-konformen Graduierung pankreatischer neuroendokriner Neoplasien und hat eine hohe prognostische Bedeutung.
- Somatostatinrezeptoren sind potenzielle therapeutische Targets und können immunhistochemisch detektiert werden.
- p53 und Rb1 helfen häufig in der Differenzierung zwischen pankreatischen neuroendokrinen Tumoren (PanNET) G3 und neuroendokrinen Karzinomen (NEC). Morphologische Kriterien sind obligat zu beachten.
- Die Differentialdiagnose zur pankreatischen neuroendokrinen Neoplasie ist breit. Immunhistochemische Untersuchungen helfen in der Typisierung.
- Molekularpathologische Untersuchungen haben aktuell einen geringen diagnostischen Stellenwert.
